# Activation of Somatostatin-Expressing Neurons in the Lateral Septum Improves Stress-Induced Depressive-like Behaviors in Mice

**DOI:** 10.3390/pharmaceutics14102253

**Published:** 2022-10-21

**Authors:** Huanhuan Li, Hyun Hailey Sung, Chunyue Geoffrey Lau

**Affiliations:** 1 Department of Neuroscience, City University of Hong Kong, Hong Kong 999077, China; 2 Shenzhen Research Institute, City University of Hong Kong, Shenzhen 518057, China

**Keywords:** neural activity, GABAergic neurons, depression, calcium imaging, limbic system

## Abstract

Depression is a debilitating mood disorder with highly heterogeneous pathogenesis. The limbic system is well-linked to depression. As an important node in the limbic system, the lateral septum (LS) can modulate multiple affective and motivational behaviors. However, the role of LS in depression remains unclear. By using c-Fos expression mapping, we first screened and showed activation of the LS in various depression-related behavioral tests, including the forced swim test (FST), tail suspension test (TST), and sucrose preference test. In the LS, more than 10% of the activated neurons were somatostatin-expressing (SST) neurons. We next developed a microendoscopic calcium imaging method in freely moving mice and revealed that LS^SST^ neural activity increased during mobility in the TST but not open field test. We hypothesize that LS^SST^ neuronal activity is linked to stress and depression. In two mouse models of depression, repeated lipopolysaccharide (LPS) injection and chronic restraint stress (CRS), we showed that LS neuronal activation was suppressed. To examine whether the re-activation of LS^SST^ neurons can be therapeutically beneficial, we optogenetically activated LS^SST^ neurons and produced antidepressant-like effects in LPS-injected mice by increasing TST motility. Moreover, chemogenetic activation of LS^SST^ neurons increased FST struggling in the CRS-exposed mice. Together, these results provide the first evidence of a role for LS^SST^ neurons in regulating depressive-like behaviors in mice and identify them as a potential therapeutic target for neuromodulation-based intervention in depression.

## 1. Introduction

The pathophysiology of depression is associated with several brain systems. Identification of the brain regions and neural circuits for depression can provide mechanistic insights that facilitate the development of therapeutics. Decades of research have made substantial progress in uncovering the neural mechanisms of depression [1,2,3]. For instance, both clinical and basic studies suggest a crucial role of the limbic system in the regulation of depression [4,5]. Structural and functional alterations in multiple regions of the limbic system have been found in patients with major depressive disorder (MDD) [6,7]. A series of studies demonstrate that limbic dysfunction in depression can be normalized by antidepressant treatment [8,9,10]. In addition, increasing evidence shows that target-specific manipulation of the limbic circuit improves depressive-like behaviors in rodents [11], yet we lack a complete understanding of how the limbic pathway contributes to depression.

The septal nucleus (or septum) is an important node of the limbic system linking the hippocampus with the hypothalamus and midbrain regions such as the ventral tegmental area (VTA) [12]. The LS is the main division of the septal nucleus. It is characterized by densely populated GABAergic inhibitory neurons [13]. Depending on the distinct anatomical location and functional connections, the LS can be further divided into three subdivisions, including the dorsal (dLS), intermediate (iLS), and ventral (vLS) regions. As each subdivision receives complex intra- and extra-septal inputs and projects to a wide range of brain regions, the function of LS remains poorly understood.

Previous studies showed that the LS is involved in multiple motivational processes and emotional states [14]. Most of these studies focused on identifying key LS pathways in regulating behaviors, such as locomotion [15,16], anxiety [17,18,19,20,21,22,23], fear [24,25,26,27,28], aggression [29,30,31,32,33,34,35], reward processing [36,37,38,39,40,41], feeding [42,43,44,45,46,47,48,49], and sociality [50,51,52,53,54,55]. However, the role of LS in depression remains unclear.

Stress is one of the most common risk factors for depression [56]. The LS has been implicated in stress responses [57]. Moreover, both clinical and animal studies suggest that LS plays a role in depression. LS hypotrophy is involved in depression, and neuronal density in the LS tends to decrease with MDD progression [58]. Animal studies have shown that antidepressant drugs can increase LS neural activity [59,60,61,62]. A recent study shows that LS GABAergic (LS^GABA^) neurons regulate depression-related behaviors through their projections to the periaqueductal gray [63]. However, it is unclear how different neuronal subpopulations of the LS^GABA^ neurons contribute to depression regulation.

In the mammalian brain, inhibitory interneurons can be subdivided by their morphology, physiological properties, and peptide/protein expression [64]. Somatostatin-expressing (SST) GABAergic neurons can provide powerful inhibitions in both local and long-range brain networks [65]. Evidence suggests that somatostatin deficits are involved in depression pathophysiology [66,67]. MDD patients show reduced SST neurotransmission [68]. Loss of SST neurons has been found in the hippocampus of individuals with bipolar disorder [69]. In the LS, SST neurons (LS^SST^) are a primary subtype of GABAergic neurons [70]. However, whether LS^SST^ neurons play a role in depression is not clear.

In this study, we investigated whether and how LS^SST^ neurons regulate depressive-like behaviors in mice. Using c-Fos expression mapping, we found that the LS was robustly recruited by multiple stressful depression-related behavioral tests. Using in vivo calcium imaging in freely behaving mice, we then identified that LS^SST^ neuronal population activity was strongly elevated during mobile states in the TST, a typical behavioral test for measuring depressive-like behaviors in mice. We next found that optogenetic activation of LS^SST^ neurons exerts antidepressant-like effects in the LPS-induced depressive-like mice. In the CRS model, chemogenetic activation of LS^SST^ neurons also reduced depressive-like behaviors in the forced swim test. Taken together, our study suggests that SST-expressing neurons in LS are an important functional component in the limbic system that regulates depressive-like behaviors in mice.

## 2. Materials and Methods

### 2.1. Animals

SST-Cre mice (013044, The Jackson Laboratory, Sacramento, CA, USA) and GCaMP6s mice (Ai162, 031562, The Jackson Laboratory, Sacramento, CA, USA) were used in this study. For the calcium imaging experiments, homozygous SST-Cre mice were crossed with heterozygous GCaMP6s mice to generate G6;SST mice. Genotyping protocols for identifying heterozygous G6;SST mice were based on the suggested protocol for GCaMP6s genotyping from the Jackson Laboratory and a previously described procedure [71]. Mice were bred and maintained at the Laboratory Animal Research Unit (LARU) under controlled environmental conditions (12 h dark/light cycle, light on/off at 08:00/20:00, food and water ad libitum). All animal experiments have been approved by the LARU and were conducted in accordance with guidelines from the Animal Research Ethics Sub-Committees of the City University of Hong Kong and the Department of Health of the Hong Kong SAR government.

### 2.2. Drugs

The clozapine N-Oxide (CNO, 34233-69-7, Tocris, Bristol, England, UK) was dissolved in vehicle (0.9% sterilized saline). Either vehicle or CNO (3 mg/kg) was administered intraperitoneally (i.p.) one hour before each behavioral test.

### 2.3. Chronic Stress-Induced Mouse Models of Depression

For the inflammation-based mouse model of depression [72], LPS (L2880, Sigma-Aldrich, St. Louis, MO, USA) was dissolved in 0.9% sterile saline (LPS working solution, 0.25 mg/mL). Adult mice were injected with LPS (0.75 mg/kg) or saline (i.p.) once daily (0.06–0.09 mL of LPS working solution or saline for 20–30 g adult mice for each injection) between 10:00 to 12:00 daily for 1 week. For the CRS-based mouse model of depression [73], mice were restrained in the ventilated 50 mL Falcon tubes (with 16 3.5 mm air vents at the wall and one 2.0 mm air vent at the nasal end of the tube) for 6 h per day during the light cycle. Mice were able to move their head and body but could not escape. During the restraint, animals had no access to food and water or social interaction. Behavioral assays were performed 24 h after the last LPS injection or restraint stress.

### 2.4. Open Field Test

The open field test (OFT) was based on a previously described procedure [74]. In brief, mice were randomly placed in one corner of the behavioral box (50 cm × 50 cm × 40 cm LWH) with light (400 lux) and allowed to explore freely for 10 min. A top camera above the box was used to record the mouse’s movement. The total distance and time spent in the center of the arena (25 cm × 25 cm) were analyzed using Toxtrac software v1.0 by tracking the mouse centroid [75].

### 2.5. Tail Suspension Test

The TST was based on a previously described procedure [76]. Briefly, mice were suspended by their tails secured with tape for 6 min in a behavior box with dim light (100 lux). Each mouse was placed in its own three-walled area without visual contact. A 3D-printed white hollow tube (4 cm length, 1.5 cm outer diameter, 1.3 cm inner diameter) was used to cover the distal part of the tail to prevent climbing during the test. A side camera was placed in the behavior box to record the mouse’s movement. Mouse speed was measured by Toxtrac software v1.0 by tracking the mouse centroid [75]. Total immobility time (speed ≤ 0.5 cm/s) or struggling time (speed > 5 cm/s) was counted during the last 4 min.

### 2.6. Forced Swim Test

The FST was based on a previously described procedure [77]. Mice were introduced to a cylindrical container (22 cm inner diameter, 20 cm depth) filled with 10 cm-deep tap water (22–24 °C) and allowed to swim for 6 min. A top camera above the behavior box was used to record the mouse’s movement. Mouse speed was analyzed by Toxtrac software v1.0 [75]. Total immobility time (speed ≤ 1.25 cm/s) or struggling time (speed > 6.25 cm/s) was counted during the last 4 min.

### 2.7. Sucrose Preference Test

For the sucrose preference test (SPT) [78], mice were habituated for 48 h to 1% sucrose water, followed by 36 h of water deprivation. On the test day, mice were placed in the behavior box with a pre-weighted bottle filled with 1% sucrose or plain water for one hour to determine their preference for 1% sucrose or water. Bottles (and fluid consumption) were weighed after the test. Sucrose preference was expressed as (sucrose intake)/(sucrose intake + water intake) × 100.

### 2.8. Stereotaxic Surgery

Mice were anesthetized with a KX mixture (Ketamine, 100 mg/kg; Xylazine, 10 mg/kg), and then head-fixed in a stereotaxic instrument (51730, Stoelting, Wood Dale, IL, USA). After incision and skull exposure, the skull surface was cleaned with sterilized PBS. For viral infusion, viruses were obtained from Addgene (AAV9-DIO-ChETA, 26968; AAV5-DIO-hM3Dq, 44361; AAV5-DIO-hM4Di, 44362, Watertown, MA, USA). Virus (AAV9-DIO-ChETA, 300 nL; AAV5-DIO-hM3Dq, AAV5-DIO-hM4Di, 500 nL) was unilaterally or bilaterally delivered into the LS (AP +1.10 mm, ML ± 0.40 mm, DV −2.80 mm) at rates of 2 nL/s using a glass pipette (1.5 µL, Drummond Wiretrol, Broomall, PA, USA) pulled by a PC-100 puller (Narishige, Tokyo, Japan). The injection pipette was left for at least 15 min before removal. An incision was sutured after surgery. Mice were monitored for at least three days after stereotaxic surgery.

For calcium imaging experiments, a gradient-index (GRIN) lens (0.5 mm diameter, 7 mm length, Mightex, Toronto, ON, Canada) coupled with a ceramic cannula (2.5 mm diameter, 1 cm length, RWD Life Science, Shenzhen, China) was slowly implanted into the left LS (AP +1.10 mm, ML +0.40 mm, DV −2.80 mm, at rates of 0.5 mm/min) of the G6;SST mice. After placement, the GRIN lens was secured with dental cement. Next, a 3D-printed lightweight head implant (2.5 g) was mounted onto the head to protect the GRIN lens implant. After implantation, mice were returned to their home cage for recovery. Care was given after the GRIN lens implant, such as daily checks and easy access to food and water.

For optogenetic experiments, an optical fiber cannula (0.2 mm diameter, 7 mm length, RWD Life Science, Shenzhen, China) was implanted approximately 200 nm above the viral injection site in the left LS (AP +1.10 mm, ML +0.40 mm, DV −2.60 mm) at rates of 0.5 mm/min. After placement, the optical fiber cannula and protector were secured with dental cement. Mice were measured with care after the implant.

### 2.9. In Vivo Ca^2+^ Imaging and Data Processing

The Ca^2+^ imaging experiments were performed 4 weeks after the GRIN lens implantation. Mice were habituated to the imaging fiber (650 µm diameter, 30,000 microfibers, 1 m length, FBR-0650-30K-100-03, Mightex, Toronto, ON, Canada) for 3 days before the behavioral test. On the test day, an additional ten-minute habitation was performed in a dim open field box (100 lux, 50 cm × 50 cm × 50 cm LWH). The imaging blue LED (470 nm excitation) power was set at 1% (0.20 mW) at the tips of the fibers. Fluorescent images were acquired at 5 Hz using the IAA software (Mightex Oasis Implant, Toronto, ON, Canada) and a cooled CCD camera (Qimaging Retiga R1, 1.4 MP, 75% peak QE, Surrey, BC, Canada).

Mightex IAA software v1.0 (Mightex, Toronto, ON, Canada) was used to extract raw GCaMP6s fluorescence of the region of interest (ROI). A background ROI fluorescence was also analyzed for each recording used as noise or fiber movement control. The GCaMP6s fluorescence acquired during the first two minutes of each recording was omitted from the analysis to minimize photobleaching. To measure the population Ca^2+^ activity during each test, dF (ΔF/F_0_) was calculated, where F_0_ was the baseline GCaMP6s fluorescence signal averaged over a one-minute window. To determine the population activity in response to specific behaviors, ΔF/F_0_ was calculated, where F_0_ was the baseline GCaMP6s fluorescence signal averaged over a 3-s window before the behavior onset. The Mightex OASIS system allowed visualization of neuronal soma in single-cell resolution. However, the signals among single neurons in LS tended to be correlated, and we averaged all activity in the field of view as population activity (akin to fiber photometry). To determine the neural activity of LS cells in response to the center visit in the OFT, dF was normalized to standard score (z score) using the GCaMP6s fluorescence acquired 3 s before and after the entry into the center zone. There was no behavior-related response in background ROI fluorescence during each recording.

### 2.10. Optogenetic Stimulation

Optogenetic experiments were performed one week after the optical fiber cannula implant. Mice were habituated to the optical fiber one day before the behavioral test battery. Additional five-minute habituation to optical fiber was performed before each behavioral test. For optogenetic photostimulation, an optical fiber was connected to a 473 nm laser controller (IOS-465, RWD Life Science, Shenzhen, China). Light output was adjusted to ~10 mW at the tip of the fibers. The stimulation protocol (5 Hz or 20 Hz or off) for each behavioral test was shown in the figures.

### 2.11. Immunohistochemistry

For the c-Fos experiments, mice were sacrificed one hour after the behavioral test. After behavioral tests, animals were deeply anesthetized with a KX mixture and were then perfused with 4% paraformaldehyde (PFA) in 1× phosphate buffered saline (PBS) at room temperature. The whole brain was post-fixed overnight in 4% PFA followed by cryoprotection in 30% sucrose in PBS for 72 h at 4 °C. Brain sections (50 µm) were prepared with a cryostat (HM525 NX, Thermo Fisher Scientific, Waltham, MA, USA). For immunohistochemistry, the sections were washed 3 times with 1X PBS for 10 min each, followed by 5% normal goat serum in 1X PBS with 0.1% Triton X-100 (X100, Sigma-Aldrich, St. Louis, MO, USA). Primary antibodies (rabbit anti-c-Fos, 1:500, ab190289; mouse anti-NeuN, 1:500, ab104224, Abcam, Cambridge, England, UK) were applied overnight at 4 °C. Next, sections were incubated with the secondary antibody (Jackson ImmunoResearch, West Grove, PA, USA) with 4′,6-diamidino-2-phenylindole (DAPI, 28718-90-3, Santa Cruz Biotechnology, Dallas, TX, USA) at room temperature for 2 h in darkness. After washing with 1X PBS, sections were mounted (Antifade mounting medium, H-1000, Vector Laboratories, Newark, CA, USA). The stained sections were stored in the dark box at 4 °C before imaging. Images were visualized by epifluorescence (Nikon Eclipse Ni-E upright fluorescence microscope, Nikon, Tokyo, Japan). Fluorescence images were quantified in ImageJ v1.53f51 (National Institutes of Health, Bethesda, MD, USA).

Viral expression, GRIN lens implant, and optical fiber positions were confirmed with immunohistochemistry. Mice with viral leakage and implant outside of the LS were removed from statistical analysis.

### 2.12. Statistical Analysis

Statistical analysis was performed using SPSS v25.0 (IBM, Armonk, NY, USA). For comparisons between data with normal distributions and equal variances, one-way analysis of variance (ANOVA) was used to check differences between multiple groups. Student’s *t*-test or Paired *t*-test was performed to test differences between the two groups. A nonparametric test was used when data did not pass tests for normality and equal variance. In the experiments related to repeated measures, repeated measures ANOVA was used for data with normal distributions and equal variances. Otherwise, Student’s *t*-test or Mann-Whitney U test was used. Unless otherwise specified, data are shown as mean ± s.e.m. Thresholds for significance were indicated as * *p* < 0.05, ** *p* < 0.01, and *** *p* < 0.001. The details of statistical tests and results were shown in the figure legends. All figures were prepared using Prism v8.0.2 (GraphPad, San Diego, CA, USA), Excel v2202 (Microsoft, Redmond, WA, USA), MATLAB R2020b (MathWorks, Natick, MA, USA), and Illustrator CC v23.0.1 (Adobe, San Jose, CA, USA).

## 3. Results

### 3.1. The LS Is Recruited by Stressful Behavioral Tests

**Figure 1:**
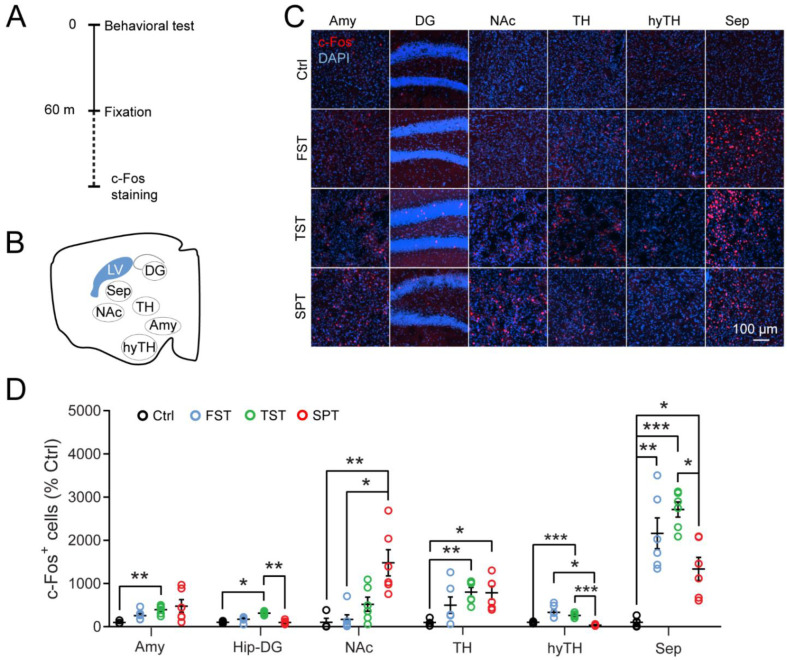
The septum responds to multiple depression-related behavioral tests. (**A**) Timeline of behavioral tests and immunostaining for c-Fos. (**B**) Schematic of brain regions for c-Fos counting. Amy: amygdala; DG: dentate gyrus; NAc: nucleus accumbens; TH: thalamus; hyTH: hypothalamus; Sep: septum; LV: lateral ventricle. (**C**) Representative images show c-Fos expression (red) in the depression-related brain regions after each behavioral test. The nuclei were stained with DAPI (blue). Scale bar, 100 µm. Ctrl, no behavioral test; TST, tail suspension test; FST, forced swimming test; SPT, sucrose preference test. (**D**) Quantification of c-Fos^+^ cells in each region. The open circle indicates the value of each mouse. Amy, Hip-DG, and NAc, Kruskal-Wallis test with Bonferroni correction. TH, LH, and Sep, One-way ANOVA followed by Dunnett T3 post hoc test. Ctrl, *n* = 4 mice for all tests and regions; TST/FST/SPT, *n* = 6 mice for all regions, except for hyTH in the SPT (*n* = 3 mice) and APC-L2 in the SPT (*n* = 5 mice). Data are mean ± s.e.m. * *p* < 0.05, ** *p* < 0.01, *** *p* < 0.001.

Evidence suggests the role of the septum in regulating affective states in rodents [12]. To investigate whether the septum is involved in the regulation of depressive-like behaviors, we first measured the expression of the neuronal activation marker c-Fos in different brain regions using three depression tests, including the TST, FST, and SPT (Figure 1A,B). Behavioral tests such as TST can be acute stressors [76]. Consistent with previous studies [79,80,81], we found that these tests recruited multiple stress and reward circuitries, such as the amygdala, hippocampus, hypothalamus, and nucleus accumbens, as shown by increases in c-Fos immunoreactivity (Figure 1C,D). Furthermore, we found robust increases of c-Fos expression in the septum following all depression-related behavioral tests relative to control mice (FST, 2162 ± 358% of control; TST, 2713 ± 175%; SPT, 1337 ± 268%; Figure 1C,D), confirming that the septum responds to depression-related behavioral tests.

**Figure 2:**
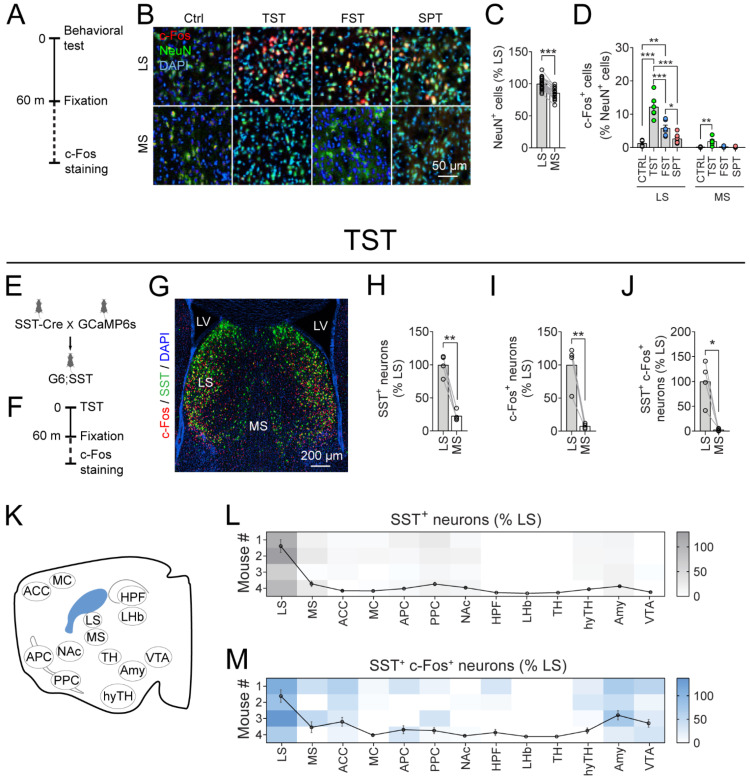
LS^SST^ neurons are strongly activated by the tail suspension test. (**A**) Timeline of behavioral tests and c-Fos immunostaining. (**B**) Representative images show c-Fos expression (red) in neurons (NeuN) of the LS and MS after each behavioral test. The nuclei were stained with DAPI (blue). Scale bar, 50 µm. (**C**) Quantification of NeuN^+^ cells in the LS and MS. Paired *t*-test. *n* = 22 mice. (**D**) Quantification of neuronal c-Fos expression in each region. The filled circle indicates the value of each mouse. LS, One-way ANOVA followed by Fisher’s Least significant difference (LSD) post hoc test; MS, Kruskal-Wallis test with Bonferroni correction. Ctrl, *n* = 4 mice; FST, TST, SPT, *n* = 6 mice. (**E**) Schematic of the generation of mice with GCaMP6s (G6) expression in the SST^+^ neurons. (**F**) Timeline of c-Fos immunostaining after the TST. (**G**) Representative image shows the c-Fos (red) expression in the GCaMP6s-expressing SST^+^ (green) neurons after TST in the septum. The nuclei were stained with DAPI (blue). LS, lateral septum; MS, medial septum; LV, lateral ventricle. Scale bar, 200 µm. (**H**–**J**) Quantification of SST^+^ neurons (**H**), c-Fos^+^ cells (**I**), and c-Fos expression (**J**) in the GCaMP6s-expressing SST^+^ neurons in the septum. Paired *t*-test. *n* = 4 mice. (**K**) Schematic of brain regions for c-Fos counting. LS: lateral septum; MS: medial septum; ACC: anterior cingulate cortex; MC: motor cortex; APC: anterior piriform cortex; PPC: posterior piriform cortex; NAc: nucleus accumbens; HPF: hippocampus; LHb: lateral habenular; TH: thalamus; hyTH: hypothalamus; Amy: amygdala; VTA: ventral tegmental area, from left to right. (**L**,**M**) Heatmap for the quantification of SST^+^ neurons (**L**) and activated SST^+^ (SST^+^c-Fos^+^) neurons (**M**) in each brain region. The line indicates mean ± s.e.m. *n* = 4 mice for each group. Data are mean ± s.e.m. * *p* < 0.05, ** *p* < 0.01, *** p < 0.001.

The neurons in LS are mainly GABAergic. SST neurons are a dominant GABAergic neuronal subtype in the LS [70]. Next, we tested whether LS^SST^ neurons responded to the TST. To label LS^SST^ neurons, we used SST neuron-targeting Cre driver line (SST-Cre) [82] and Ai162 (GCaMP6s) mice, a newly developed Cre-dependent TIGRE2.0 reporter line expressing the GFP-based, genetically encoded calcium indicator GCaMP6s [71]. We crossed the SST-Cre mice with GCaMP6s mice, resulting in the expression of green fluorescent protein (GFP) in the somatostatin-expressing neurons (G6;SST; Figure 2E,G). The LS showed a higher density of SST neurons than the MS (22.8 ± 4.0% of LS; Figure 2G,H). Consistent with the above-mentioned results (Figure 2D), there was more c-Fos activation in the LS neurons than in the MS (7.6 ± 1.8% of LS) after TST (Figure 2F,G,I). There were more activated SST neurons in the LS compared to MS (2.8 ± 1.2% of LS; Figure 2J). In addition, the LS showed the highest density of SST^+^ neurons and SST activation among all the counted regions (Figure 2K–M). Taken together, these results demonstrate that the LS^SST^ neurons are activated by behavioral tests commonly used to assess depressive-like behaviors.

### 3.2. LS^SST^ Neurons Are Active during TST but Not OFT Mobility

**Figure 3:**
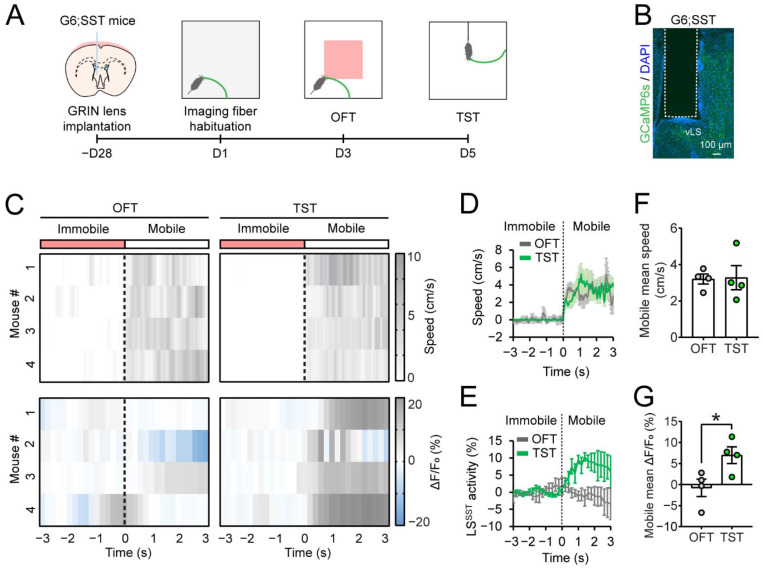
The LS^SST^ neurons increase their activity during TST mobility but not OFT mobility. (**A**) Schematic of GRIN lens implant in the LS of G6;SST mice and timeline of behavioral testing. (**B**) Representative anatomical location of GRIN lens implant in the LS. GCaMP6s-expressing SST^+^ neurons, green; DAPI, blue. Scale bar, 100 µm. (**C**) Heatmap of mouse speed (**top**) and LS^SST^ activity (**bottom**) during the OFT (**left**) and TST (**right**). The dotted line indicates mobile onset. (**D**,**E**) Normalized mouse speed (**D**) and LS^SST^ activity (**E**) in the OFT and TST as shown in (**C**). The error bars represent s.e.m. OFT, gray line; TST, blue line. (**F**,**G**) Average mouse speed (**F**) and LS^SST^ activity (G) during the OFT mobility and TST mobility. The filled circle indicates data from each mouse. Student’s *t*-test. *n* = 4 mice for each group. Data are mean ± s.e.m. * *p* < 0.05.

To directly probe the role of LS^SST^ neurons in regulating depressive-like behaviors, we developed an imaging method using microendoscopic calcium imaging to measure the activity of LS^SST^ neurons in freely moving mice during behavioral tests. After the generation of the G6;SST mice, we implanted a GRIN lens into the left LS, which projected light collected from the brain to an optical fiber (0.59 NA) and captured by a CCD camera (Figure 3A,B). In the TST, we found that LS^SST^ neurons were robustly activated during mobility (Video S1). To assess the role of LS^SST^ neurons during mobility, we compared the population activity of LS^SST^ neurons in response to mobile states in either OFT or TST (Figure 3A,C). LS^SST^ population activity was significantly higher when the mice were mobile in the TST than in the OFT (OFT, –0.79 ± 2.1%; TST, 7.0 ± 2.0%; Figure 3C,E,G). Notably, there was no difference in speed observed during the motile state comparing the OFT and TST (OFT, 3.2 ± 0.28 cm/s; TST, 3.3 ± 0.7 cm/s; Figure 3C,D,F). In addition, LS^SST^ population activity remained unchanged whether mice were in the center zone vs. along the walls in the OFT (Figure S1). Together, these data strongly suggest that the increased LS^SST^ population activity depends on behavioral states rather than locomotion. These findings show that the LS^SST^ neurons are specifically involved in mobile behavior during TST, which is related to stress.

### 3.3. Optogenetic Activation of LS^SST^ Neurons Decreases Depressive-Like Behaviors Induced by LPS in the TST

**Figure 4:**
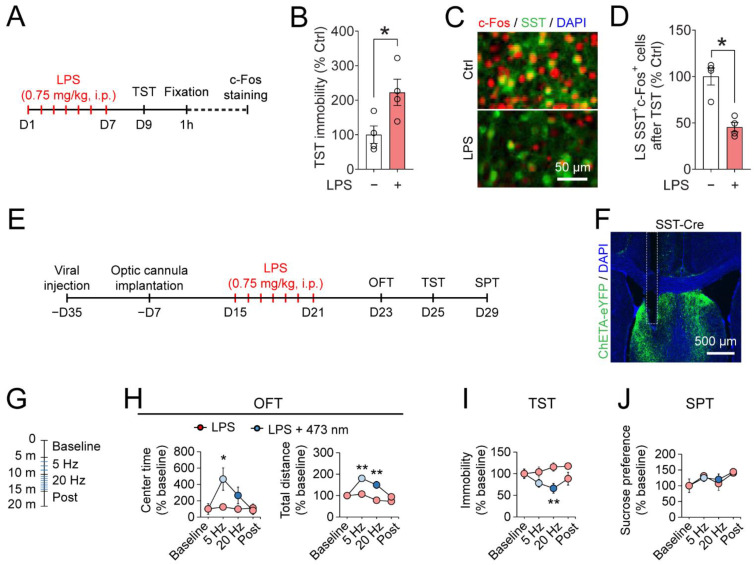
Optogenetic activation of LS^SST^ neurons reduces LPS-induced depressive-like behaviors in the tail suspension test. (**A**) Timeline of LPS injection, TST, and c-Fos immunostaining. (**B**) Quantification of mouse immobility in the TST. Student’s *t*-test. *n* = 4 mice for each group. (**C**) Representative image shows c-Fos expression (red) in the LS^SST^ neurons (green). The nuclei were stained with DAPI (blue). Scale bar, 50 µm. (**D**) Quantification of SST^+^c-Fos^+^ neurons in the LS. Mann-Whitney U test. *n* = 4 mice for each group. (**E**) Timeline of viral injection, optical cannula implant, LPS injection, and behavioral testing. (**F**) Representative image shows ChETA expression and anatomical location of optical fiber implant in the LS. Scale bar, 500 µm. (**G**) Schematic of the optogenetic stimulation protocol in the OFT, TST, and SPT. (**H**) Normalized center time (**left**) and total distance (**right**) in the OFT after LPS injections. OFT center time: 5 Hz and 20Hz, Student’s *t*-test; post, Mann-Whitney U test; OFT total distance, Two-way repeated measures ANOVA followed by LSD post hoc test. (**I**) Normalized immobility time in the TST after LPS injections. Student’s *t*-test. (**J**) Normalized sucrose preference in the SPT. 5 Hz, Student’s *t*-test; 20 Hz and post, Mann-Whitney U test. LPS, *n* = 6 mice; LPS + 473 nm, *n* = 7 mice. Data are mean ± s.e.m. * *p* < 0.05, ** *p* < 0.01.

LPS has been widely used in modeling aspects of depressive-like symptoms in rodents [72]. Next, we established a well-validated mouse model of depression by intraperitoneal injection of LPS (0.75 mg/kg) for one week (Figure 4A). LPS-injected mice showed increased immobility time in the TST (LPS, 222.5 ± 38.1% of saline-injected control; Figure 4B). Repeated LPS injection also significantly reduced the number of activated SST neurons in the LS one hour after the TST (LPS, 45.4 ± 5.1% of saline-injected control; Figure 4C,D). The results establish the repeated LPS injection as an inflammation-based model for studying behavioral changes and neuropathology related to depression.

Since LS^SST^ neuronal activity was reduced by LPS, we next asked whether activation of LS^SST^ neurons can affect depressive-like behaviors in the LPS model of depression. The AAV9-DIO-ChETA virus was unilaterally injected into the LS of SST-Cre mice (Figure 4E). Four weeks after viral expression, an optical fiber was implanted above the injection site of the LS (Figure 4E,F). After one week of recovery from surgical procedures, mice were subjected to repeated LPS injections (Figure 4E,F). Mice were subjected to a battery of behavior tests, and performance was measured in the same mouse with or without optogenetic activation (Figure 4E). In the OFT, 5 Hz (20-ms pulse width, for five minutes) photostimulation (473 nm) markedly and significantly increased, while 20 Hz (5-ms pulse width, for five minutes) stimulation modestly but insignificantly increased, the duration of center time in LPS mice compared to no light stimulation control. No change in center time was found post-photostimulation (LPS no light vs. LPS light on: 5 Hz, 124.1 ± 36.6% to 465.6 ± 136.3%; 20 Hz, 98.1 ± 37.0% to 265.6 ± 102.4%; post, 111.3 ± 51.4% to 81.3 ± 49.0%; Figure 4G,H). In addition, the total distance in the LPS-injected mice was significantly increased by both 5 Hz and 20 Hz light activations of the LS^SST^ neurons, suggesting some anxiolytic-like effect (LPS no light vs. LPS light on: 5 Hz, 107.0 ± 13.9% to 180.1 ± 13.7%; 20 Hz, 78.1 ± 8.1% to 149.9 ± 16.4%; post, 73.1 ± 11.8% to 94.1 ± 6.9%; Figure 4G,H). In the TST, 20 Hz light stimulation strongly and significantly, while 5 Hz stimulation moderately but insignificantly, deceased the immobility time (LPS no light vs. LPS light on: 5 Hz, 104.1 ± 11.5% to 78.1 ± 9.4%; 20 Hz, 115.5 ± 9.9% to 88.5 ± 14.8%; post, 117.6 ± 6.6% to 88.5 ± 14.8%; Figure 4G,I). Optogenetic activation of LS^SST^ neurons did not influence the SPT sucrose preference in the LPS-injected mice (LPS no light vs. LPS light on: 5 Hz, 131.0 ± 6.5% to 125.0 ± 11.5%; 20 Hz, 106.9 ± 21.7% to 119.9 ± 17.4%; post, 140.5 ± 5.1% to 144.2 ± 5.1%; Figure 4G,J).

As a control, we next examined the behavioral effects of optogenetic activation of LS^SST^ neurons in the healthy control (unstressed) mice (Figure S2A,B). Optogenetic activation of LS^SST^ neurons was performed in two different patterns: 5 Hz, 20-ms pulse width, for ten minutes; 20 Hz, 5-ms pulse width, for ten minutes (total light exposure is the same for the two stimulation paradigms; Figure S2C). Activating LS^SST^ neurons did not affect OFT center time (No light vs. light on: 5 Hz, 89.5 ± 30.5% to 124.3 ± 44.5%; 20 Hz, 54.3 ± 52.3% to 59.2 ± 19.4%) or total distance traveled (No light vs. light on: 5 Hz, 69.6 ± 10.0% to 78.2 ± 4.8%; 20 Hz, 43.6 ± 9.7% to 473 nm: 64.8 ± 6.3%; Figure S2D), suggesting that activation of the LS^SST^ neurons has little effect on the anxious levels and general locomotive functions in control mice.

We then performed photostimulation of the LS^SST^ neurons during TST (Figure S2E). Both 5 Hz and 20 Hz light stimulation modestly but significantly decreased the TST immobility time compared to no-light control (No light vs. light on: 5 Hz, 144.9 ± 5.7% to 116.9 ± 11.3%; 20 Hz, 151.1 ± 6.0% to 114.0 ± 8.7%; post, 143.2 ± 3.0% to 174.6 ± 14.3%; Figure S2F). In the SPT, 20 Hz but not 5 Hz photostimulation led to an increase in sucrose preference (No light vs. light on: 5 Hz, 178.9 ± 45.7% to 137.5 ± 23.5%; 20 Hz, 76.0 ± 30.4% to 182.8 ± 15.2%; post, 191.8 ± 26.0% to 181.2 ± 9.3%; Figure S2G). These data demonstrate that activation of LS^SST^ neurons had some mild effects on depressive-like behaviors in control mice.

Taken together, these results suggest that activation of LS^SST^ neurons can improve depressive-like behaviors in LPS-injected mice.

### 3.4. Chemogenetic Activation of the LS^SST^ Neurons Reduces Depressive-like Behaviors Induced by CRS in the FST

**Figure 5:**
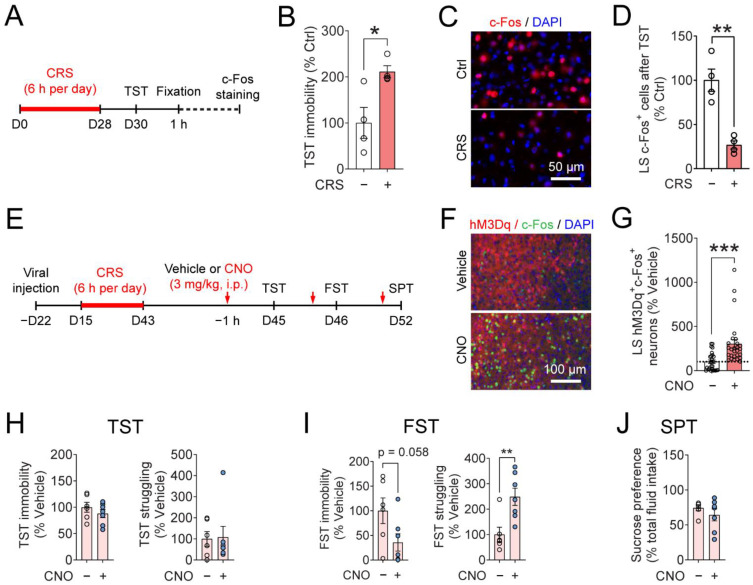
Chemogenetic activation of LS^SST^ neurons improves CRS-induced depressive-like behaviors in the forced swim test. (**A**) Timeline of CRS protocol, TST, and c-Fos immunostaining. (**B**) Quantification of mouse immobility in the TST. Mann-Whitney U test. *n* = 4 mice for each group. (**C**) Representative image shows c-Fos expression (red) in the LS. The nuclei were stained with DAPI (blue). Ctrl: control; CRS: chronic restraint stress. Scale bar, 50 µm. (**D**) Quantification of c-Fos^+^ neurons in the LS. Student’s *t*-test. *n* = 4 mice for each group. (**E**) Timeline of viral injection, CNO administration, CRS protocol, and behavioral testing. (**F**) Representative images showed c-Fos expression (green) in the hM3Dq-expressing neurons in the LS of SST-Cre mice. Scale bar, 100 µm. (**G**) Quantification of hM3Dq^+^ c-Fos^+^ neurons in the LS. Mann-Whitney U test. Vehicle: *n* = 26 sections from 9 mice; CNO: *n* = 27 sections from 9 mice. (**H**) Quantification of TST immobility (**left**) and struggling (**right**) time after CRS. TST immobility, Student’s *t*-test; TST struggling, Mann-Whitney U test. (**I**) Quantification of FST immobility (**left**) and struggling (**right**) time after CRS. Student’s *t*-test. (**J**) Quantification of mouse sucrose preference in the SPT after CRS. Mann-Whitney U test. *n* = 9 mice for each group. Data are mean ± s.e.m. The filled circle indicates the value of each mouse. * *p* < 0.05, ** *p* < 0.01, *** *p* < 0.001.

Our previous results demonstrated that acute, optogenetic activation of LS^SST^ neurons can alleviate depressive-like behaviors in mice injected with LPS (Figure 4), but it is unclear whether slower (minutes to hours) activation can also afford similar beneficial effects. In the CRS-induced mouse model of depression, we first found that the density of TST-induced c-Fos expression was significantly lower in the CRS mice (26.8 ± 4.7% of Ctrl) compared to the control mice (Figure 5A–D). This led us to hypothesize that elevating LS neural activity can be therapeutically beneficial to depressive-like behaviors.

To evaluate the effects of chemogenetic activation of LS^SST^ neurons on mouse depressive-like behaviors, we used designer receptors exclusively activated by a designer drug (DREADD). We bilaterally delivered a Cre-dependent AAV5 encoding hM3Dq fused with mCherry (AAV5-DIO-hM3Dq) into the LS of the SST-Cre mice (Figure 5E,F; Figure S3A,B). Three weeks after injection, mouse depressive-like behaviors were measured (Figure 5E; Figure S3B). To activate the LS^SST^ neurons, the designer drug CNO was injected (3 mg/kg, i.p.) one hour before each behavioral test (Figure 5E–G; Figure S3B). In healthy controls, CNO injection did not significantly alter TST immobility and struggling (immobility, CNO: 65.4 ± 9.2% of vehicle; struggling, CNO: 190.3 ± 46.7% of vehicle; Figure S3C), FST immobility and struggling (immobility, CNO: 109.8 ± 15.0% of vehicle; struggling, CNO: 104.7 ± 16.8% of vehicle; Figure S3D), or sucrose preference (vehicle, 68.7 ± 10.2%; CNO, 49.1 ± 8.1%; Figure S3E). These results suggest that CNO injection and hM3Dq activation did not induce behavioral side effects in healthy, unstressed mice.

We next probed whether the chemogenetic activation of LS^SST^ neurons can affect behavioral changes induced by CRS. CNO injection had no effect on either the TST immobility (103.7 ± 8.5% of vehicle) or struggling (102.6 ± 42.8% of vehicle) in the CRS mice (Figure 5H). In the FST, injection of CNO moderately but insignificantly decreased the immobility time (41.1 ± 14.5% of vehicle) while it significantly increased the struggling time (241.6 ± 27.8% of vehicle) in the CRS mice (Figure 5I). In the SPT, CRS mice that received CNO injection did not show changes in sucrose preference (vehicle, 67.8 ± 5.3%; CNO, 61.8 ± 7.7%; Figure 5J). These results indicate that activation of LS^SST^ neurons reduces depressive-like behaviors induced by chronic restraint stress.

### 3.5. Inhibiting LS^SST^ Neurons Does Not Induce Depressive-like Behaviors in the Unstressed Mice

**Figure 6:**
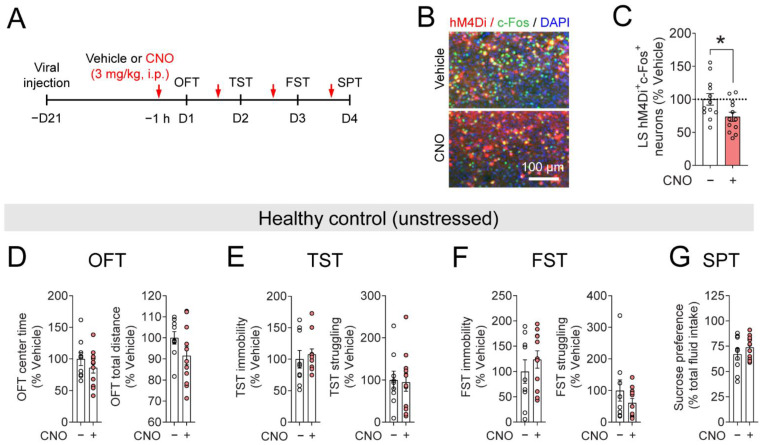
Inhibiting LS^SST^ neurons is insufficient to induce depressive-like behaviors in unstressed mice. (**A**) Timeline of viral injection, CNO administration, and behavioral testing. (**B**) Representative images showed c-Fos expression (green) in the hM4Di-expressing neurons in the LS of SST-Cre mice. Scale bar, 100 µm. (**C**) Quantification of hM4Di^+^c-Fos^+^ neurons in the LS. Student’s *t*-test. *n* = 12 sections from 4 mice for each group. (**D**) Quantification of mouse center time (**left**) and total distance (**right**) in the OFT. Student’s *t*-test. (**E**) Quantification of mouse immobility and struggling in the TST. Immobility, Mann-Whitney U test; struggling, Student’s *t*-test. (**F**) Quantification of mouse immobility and struggling in the FST. Immobility, Student’s *t*-test; struggling, Mann-Whitney U test. (**G**) Quantification of mouse sucrose preference in the SPT. Student’s *t*-test. Vehicle, *n* = 9 mice; CNO, *n* = 11 mice. Data are mean ± s.e.m. The filled circle indicates the value of each mouse. * *p* < 0.05.

As chemogenetic activation of LS^SST^ neurons afforded alleviation of depressive-like behaviors (Figure 5), we wondered whether suppressing them can induce depressive-like behaviors in healthy control (unstressed) mice. Mice were bilaterally injected with a Cre-dependent AAV5 encoding hM4Di fused with mCherry (AAV5-DIO-hM4Di) into the LS of the SST-Cre mice (Figure 6A–C). We then evaluated mouse depressive-like behaviors in a battery of behavioral tests (Figure 6B). For the inhibition of LS^SST^ neurons, CNO was injected (3 mg/kg, i.p.) one hour before each behavioral test (Figure 6A–C). In the OFT, injection of CNO induced small and insignificant reductions in the center time (86.2 ± 8.6% of vehicle) and total distance (91.5 ± 4.3% of vehicle) in the unstressed mice (Figure 6D). TST immobility (107.5 ± 8.8% of vehicle) and struggling (94.7 ± 21.3% of vehicle) were not influenced by the CNO-mediated inhibition of LS^SST^ neurons (Figure 6E). Mice injected with CNO exhibited increased FST immobility (123.7 ± 17.0% of vehicle) and decreased FST struggling (60.9 ± 14.0% of vehicle), but they did not reach significance (Figure 6F). In the SPT, no difference in sucrose preference was observed between the two groups (vehicle: 67.1 ± 5.8%, CNO: 73.8 ± 3.4%; Figure 6G). Thus, these results demonstrate that inhibition of LS^SST^ neurons was insufficient to induce depressive-like behaviors in healthy, unstressed mice.

## 4. Discussion

The septum is an evolutionarily conserved part of the limbic system that has diverse functions in regulating emotional states and behavioral responses, such as anxiety, locomotion, feeding, and sociality [12,14,83]. Previous studies suggest that the lateral septum is associated with depression [63,84,85], but the underlying neural substrates are less well-understood. Our results presented here show that SST neurons in the LS contribute to the regulation of depressive-like behaviors in mice. We found that classical depression-related behavioral tests robustly recruit the LS rather than MS. Subsequent calcium imaging revealed that LS^SST^ neurons drive mobile states in the TST. Optogenetic and chemogenetic experiments showed that activation of the LS^SST^ neurons produces anti-depressive like effects in stress-induced, depressive-like mice. However, inhibiting LS^SST^ neurons does not induce depressive-like behaviors in healthy mice. Together, these results establish a previously unknown role for LS^SST^ neurons in depression, providing further evidence for the LS as an essential limbic component in this disorder.

SST neurons are important for many brain functions, including sensory perception, movement, motivation, reward, and affective states [65]. Reduced SST in the brain has been consistently reported in patients with depression [66,86,87]. Thus, understanding the functions of SST neurons may provide mechanistic insight into the neurobiology of depression. Early anatomical studies show that SST neurons are a conserved cell population in the septum across species, including human [88,89], rodents [90,91,92], birds [93], frogs [94], and reptiles [95,96]. Here, we show that a subset of SST neurons in the LS is activated during the mobility state in TST. In vivo calcium imaging demonstrates that LS^SST^ calcium activity is increased during TST mobility, suggesting that the LS^SST^ neuronal activity may be an important neural correlate of immobile behavior, which is exacerbated in depressive-like mice. Importantly, LS^SST^ neurons specifically respond to TST mobility, but they do not respond to general locomotion during the OFT. These results show that the TST can be, at the same time, both a stressor acting on the mouse and behavioral output. Since both are related to depressive-like behaviors, future works are needed to interrogate how LS^SST^ neurons contribute to these behavioral states. In addition, we found different patterns of LS^SST^ neuronal population activity during the OFT and TST (Figure 3). Most notably, there were differences in calcium dynamics among different recorded animals in our in vivo calcium recording data. For instance, the LS^SST^ neuronal population activity of one mouse was increased after the TST mobile onset but decreased shortly afterward (mouse #2; Figure 3C). There are a few possibilities that might explain these LS^SST^ neuronal calcium fluctuations. One potential explanation is the temporal activation of LS^SST^ neurons may be different in response to behavior such as motor activity. Although LS^SST^ neuronal somata and neuropils showed synchronized calcium transients during mobility in the TST (Video S1), we did not address how LS^SST^ neurons correlate with animal movements in the OFT and TST, such as mobility onset, speed, acceleration, and movement termination. Future investigations are needed to determine LS^SST^ neuron activity at the single-cell resolution with better imaging techniques, which will provide fundamental insights into the relationship between LS^SST^ neuron activity and animal behavior during depression-related behavioral tests.

Our chemogenetic and optogenetic experiments demonstrated that activation of LS^SST^ neurons reduces stress-induced depressive-like behaviors. These findings support the hypothesis that SST neurons in the LS are involved in depression. Given that inhibition of LS^SST^ neurons did not induce depressive-like behaviors in the healthy control mice, it is necessary to investigate further whether dysfunctions of LS^SST^ neurons contribute to the pathogenesis of depression. Currently, less is known about the changes of LS^SST^ neurons in animal models of depression and how antidepressant treatment affects their activity. Resolving these questions may provide further evidence for the LS^SST^ neurons in depression. A recent study shows that vesicular GABA transporter (VGAT)-positive GABAergic neurons in the LS exhibit increased c-Fos activation after chronic unpredictable stress. Activation of LS^GABA^ neurons and their projections to the periaqueductal gray increase depressive-like behaviors, while inhibiting this LS^GABA^ circuit reduces depression phenotypes in mice [63]. Our results that activation of LS^SST^ neurons alleviates depressive-like behaviors are not contradictory to the findings of Wang et al. (2021) due to the heterogeneity and complexity of neuronal subtypes. SST-expressing neurons only account for about 20% of the LS^GABA^ neurons, meaning that the LS contains many other GABAergic neuron subtypes. Moreover, LS contains a fraction of glutamatergic excitatory neurons [12,97]. The architecture and functions of LS local circuits are not clear. The SST neurons in the septum have reciprocal connections with both pyramidal and inhibitory neurons in the hippocampus, and different circuits produce different behavioral effects [90,98,99,100,101,102]. LS^SST^ neurons are in part innervated by the pyramidal neurons in the hippocampal CA3, and this CA3 to LS^SST^ pathway can directly regulate depressive-like behaviors [103]. In addition, SST neurons in the LS send projections to the lateral hypothalamus, which can regulate gamma oscillation and foraging behavior in mice [104]. A recent study demonstrates that SST neurons in the dorsal LS and their subcortical targets can control fear responses [28]. In this study, we found that optogenetic activation of the LS^SST^ neurons increased OFT center time and decreased TST immobility in the LPS-injected mice. Moreover, chemogenetic activation of the LS^SST^ neurons increased FST struggling time in the CRS-exposed mice. However, neither optogenetic nor chemogenetic activation of LS^SST^ neurons affected sucrose preference in mice exposed to LPS injections and CRS, respectively. These results indicate that LS^SST^ neurons may be associated with the regulation of specific depressive-like behaviors in mice, especially behavioral despair. Clinically, patients with depression experience diverse symptoms, such as despair, loss of pleasure, and anxiety [2]. One hypothesis is that these depressive symptoms are related to changes in different neural circuits regulating behaviors in response to stress and reward [4]. For example, dysfunction of the hypothalamic-pituitary-adrenal (HPA) axis is involved in maladaptive responses to stress such as behavioral despair, circuit deficits in the reward system contribute to anhedonia, and changes in the amygdala may result in anxiety-like symptoms [1,3,5]. Thus, it would be interesting to dissect the function of LS in various behavioral phenomena such as stress handling, motivation, and motor control. Therefore, future studies focused on the connectivity of LS^SST^ neurons, either intra-septal microcircuits or extra-septal long-range circuits, are indispensable for elucidating mechanisms that underlie the role of LS^SST^ neurons in depression regulation.

Substantial evidence shows that the LS is an essential node in the limbic system controlling stress responses. Previous studies indicate that the LS is activated by acute stress [80,105,106,107]. It has been shown that VGAT-expressing LS^GABA^ neurons respond to acute stressors, including loud sound, light, and water spray [47]. Another study demonstrates that the calcium activity of neurotensin-expressing cells, another common subtype of GABAergic neurons in the LS (more ventrally distributed), is increased in response to several acute stressors, such as tail suspension, predatory cues, and aversive electric shocks [108]. Consistent with these findings, our data show that ~15% of LS^SST^ neurons are recruited by the TST, a behavioral task that includes multiple acute stressors, such as tail suspension and novel environment. Moreover, LS hypofunction has been found in depressive-like mice subjected to chronic stress [54,85], consistent with our results. Here, we used two chronic stressors (repeated low-dose LPS intraperitoneal injection and restraint) to induce depressive-like behaviors in mice. Chronic, rather than acute, stress is a major risk factor for the pathogenesis of depression [56]. Increased inflammation has also been implicated in the etiology of depression [2]. Repeated immune activation by LPS can induce depressive-like behaviors in mice [72]. In this study, we measured the behavioral changes induced by LPS one day after the last injection to minimize the effect of LPS-induced sickness behaviors. Indeed, LPS-injected mice exhibited increased immobility in the TST (Figure 4B). Correlating with LPS-induced depressive-like behaviors, LS^SST^ neurons showed c-Fos activation in this inflammation-based mouse model of depression (Figure 4C,D), suggesting chronic stress exposure could result in dampened LS^SST^ neuronal activity. In line with this finding, we found that CRS-induced depressive-like mice showed increased TST immobility and reduced c-Fos expression in the LS. Unlike the induction of depressive-like behaviors by LPS-mediated inflammation, chronic restraint stress promotes the development of depressive-like behaviors by recapitulating the stressful experience during daily life, which may induce a myriad of neurochemical changes in multiple brain regions [56,73]. These results suggest that altered neuronal activity in the LS may be a specific feature of behavioral despair in depressive-like mice. Consequently, using two different methods, namely optogenetics and chemogenetics, we demonstrated that the elevation of LS^SST^ neurons enhances struggling behavior in depressive-like mice, suggesting an alleviation of learned helplessness behavior in depression. In our current study, one limitation is the lack of cellular and molecular mechanisms underlying the activation and suppression of LS neuronal activity in response to acute and chronic stressors, respectively. It would be important to decipher how chronic stress can lead to long-term changes in circuit function and perhaps neuroinflammation that contributes to depressive-like behaviors.

## 5. Conclusions

In summary, our results indicate that LS^SST^ neurons are a subcortical neural substrate in the limbic region that contributes to the regulation of depressive-like behaviors in mice. The activity of LS^SST^ neurons may serve as an important neural correlate of depression. An interesting topic for further study is how LS^SST^ neurons engage in intra- and extra-septal circuit function. It would be of fundamental importance to identify whether these LS^SST^ pathways contribute to regulating stress and depressive-like behaviors, which will extend our understanding of the circuit mechanisms underlying depression regulation by the limbic system. The lateral septum may be a promising limbic site for neuromodulation or deep brain stimulation in developing intervention strategies for depression.

## Data Availability

All data in this study are available from the corresponding author on reasonable request.

## Supplementary Materials

The following supporting information can be downloaded at: https://www.mdpi.com/article/10.3390/pharmaceutics14102253/s1, Figure S1: LS^SST^ neuronal activity does not correlate with thigmotaxis in the OFT; Figure S2. Behavioral effects of optogenetic activation of LS^SST^ neurons on the depressive-like behaviors of the unstressed mice; Figure S3. Behavioral effects of chemogenetic activation of LS^SST^ neurons on the depressive-like behaviors of the unstressed mice; Video S1: LS^SST^ activity during the tail suspension test.
