# Supplementary material for: Activation of Somatostatin-Expressing Neurons in the Lateral Septum Improves Stress-Induced Depressive-like Behaviors in Mice
Source: Pharmaceutics. 2022 Oct 21;14(10):2253. doi: 10.3390/pharmaceutics14102253 (PMC9607457; doi:10.3390/pharmaceutics14102253)
Supplement: Supplementary file 1 [file pharmaceutics-14-02253-s001.zip › pharmaceutics-1970681-supplementary.pdf]

pharmaceutics

Supplementary Materials: Activation of Somatostatin-Expressing Neurons in the Lateral Septum Improves Stress-Induced Depressive-Like Behaviors in Mice

Huanhuan Li [1,2], Hyun Hailey Sung [1,2] and Chunyue Geoffrey Lau [1,2,*]

[1] Department of Neuroscience, City University of Hong Kong, Hong Kong 999077, China
[2] Shenzhen Research Institute, City University of Hong Kong, Shenzhen 518057, China
* Correspondence: geoff.lau@cityu.edu.hk; Tel.: +852-3442-4345

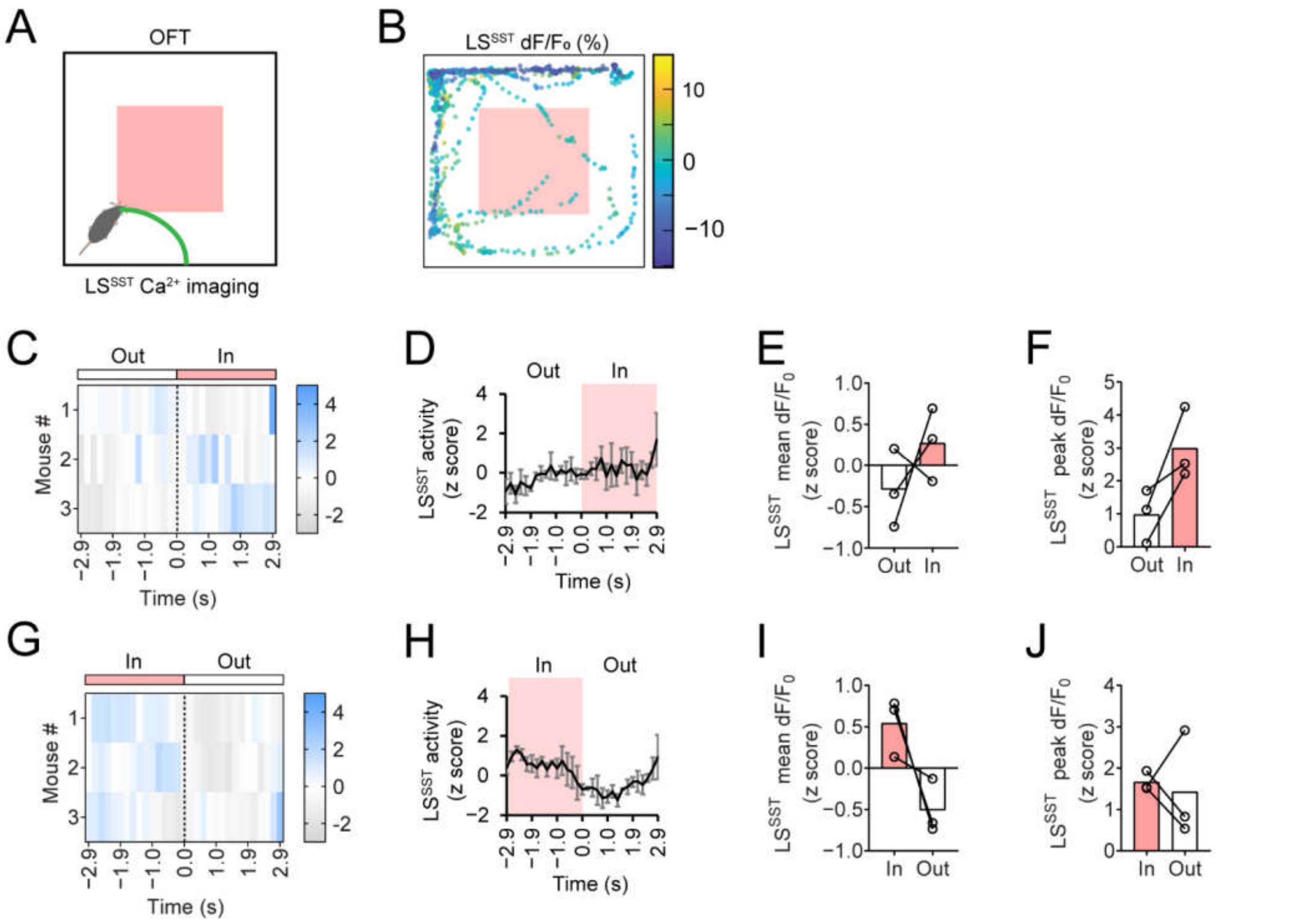

Figure S1. LS^{SST} neuronal activity does not correlate with thigmotaxis in the OFT. (**A**) Schematic of calcium recording in the LS^{SST} neurons of the G6;SST mice during OFT. The red area indicates center zone. (**B**) Representative spatial heatmap for the LS^{SST} population activity during the OFT. (**C**) Heatmap of the LS^{SST} population activity before and during center entry. The dotted line indicates entry into the center. (**D**) Averaged LS^{SST} activity (z score) during center entries as shown in (**C**). The red area indicates center entry. The error bars represent s.e.m. (**E,F**) Average LS^{SST} mean (**E**) and peak activity (**F**). The filled circle indicates data from each mouse. Student's t-test. (**G**) Heatmap of the LS^{SST} population activity before and after center leave. The dotted line indicates entry into the center. (**H**) Averaged LS^{SST} activity (z score) as shown in (**G**)**.** The red area indicates center entry. The error bars represent s.e.m. (**I,J**) Average LS^{SST} mean (**I**) and peak activity (**J**). The filled circle indicates data from each mouse. Student's t-test. The LS^{SST} neurons did not show significant changes related to the anxiety-like behaviors in the OFT. n = 3 mice. Data are mean ± s.e.m.

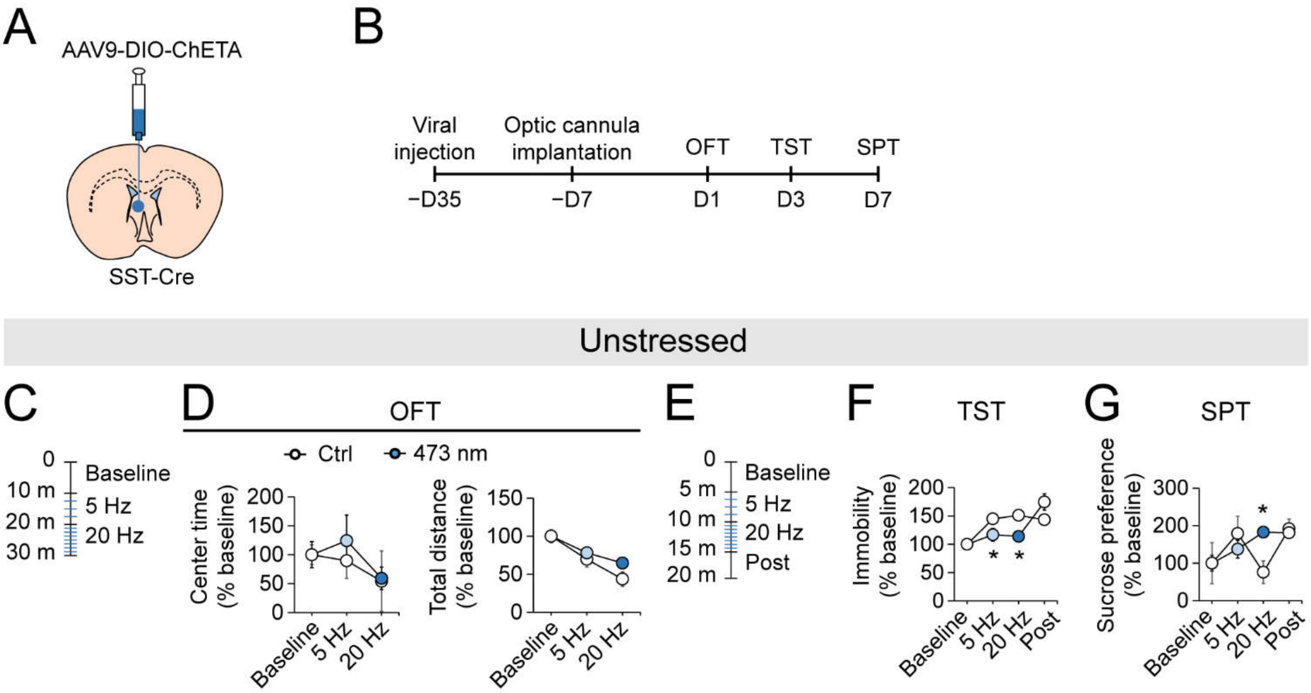

Figure S2. Behavioral effects of optogenetic activation of LS^{SST} neurons on the depressive-like behaviors of the unstressed mice. (**A**) Schematic of unilateral viral injection of Cre-dependent AAV9-DIO- ChETA into the LS of SST-Cre mice. (**B**) Timeline of viral injection, optic cannula implant, and behavioral testing. (**C**) Schematic of the optogenetic stimulation protocol in the OFT. (**D**) Normalized center time (left) and total distance (right) in the OFT. OFT center time, Mann-Whitney U test; OFT total distance, Two-way repeated measures ANOVA followed by LSD post hoc test. (**E**) Schematic of the optogenetic stimulation protocol in the TST and SPT. (**F**) Normalized immobility time in the TST. Student's t-test. (**G**) Normalized sucrose preference in the SPT. 5 Hz and post, Student's t-test; 20 Hz, Mann-Whitney U test. Ctrl, n = 3 mice; 473 nm, n = 10 mice. Data are mean ± s.e.m. * $p < 0.05$.

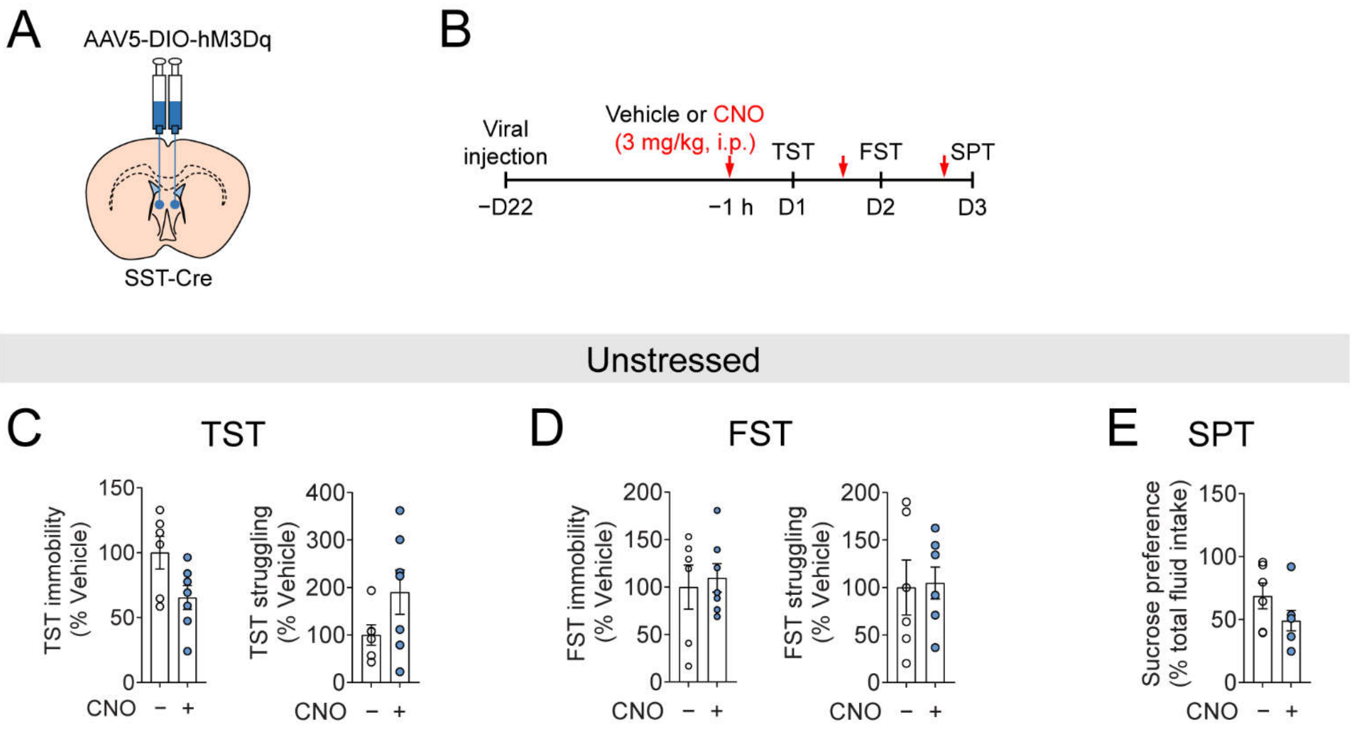

Figure S3. Behavioral effects of chemogenetic activation of LS^{SST} neurons on the depressive-like behaviors of the unstressed mice. (**A**) Schematic of bilateral viral injection of Cre-dependent AAV5-DIO-hM3Dq into the LS of SST-Cre mice. (**B**) Timeline of viral injection, CNO administration, and behavioral testing. (**C**) Quantification of mouse immobility (left) and struggling (right) time in the TST. Student's t-test. (**D**) Quantification of mouse immobility (left) and struggling (right) time in the FST. Student's t-test. (**E**) Quantification of mouse sucrose preference in the SPT. Student's t-test. Vehicle, $n = 6$ mice; CNO, $n = 7$ mice. Data are mean ± s.e.m. The filled circle indicates the value of each mouse.
